# Associations between high triglycerides and arterial stiffness in a population-based sample: Kardiovize Brno 2030 study

**DOI:** 10.1186/s12944-020-01345-0

**Published:** 2020-07-15

**Authors:** Iuliia Pavlovska, Sarka Kunzova, Juraj Jakubik, Jana Hruskova, Maria Skladana, Irma Magaly Rivas-Serna, Jose R. Medina-Inojosa, Francisco Lopez-Jimenez, Robert Vysoky, Yonas E. Geda, Gorazd B. Stokin, Juan P. González-Rivas

**Affiliations:** 1 International Clinical Research Center (ICRC), St. Ann’s University Hospital Brno | FNUSA-ICRC, Brno, Czech Republic; 2 Department of Public Health, Faculty of Medicine, Masaryk University, Brno, Czech Republic; 3 Division of Preventive Cardiology, Department of Cardiovascular Medicine, Mayo Clinic, Rochester, MN USA; 4 Department of Neurology, Barrow Neurological Institute, Phoenix, USA; 5 Department of Global Health and Population | Harvard T.H. Chan School of Public Health, Boston, USA

**Keywords:** Triglycerides, Vascular stiffness, Metabolic syndrome, Cardio-ankle vascular index, Atherosclerosis, Risk factors

## Abstract

**Background:**

The term arterial stiffness (ArSt) describes structural changes in arterial wall related to the loss of elasticity and is known as an independent predictor of cardiovascular diseases (CVD). The evidence relating to ArSt and triglycerides (TG) shows contradictory results. This paper means to survey the association between high TG and ArSt, utilizing the cardio-ankle vascular index (CAVI).

**Methods:**

Subjects aged between 25 and 64 years from a random population-based sample were evaluated between 2013 and 2016. Data from questionnaires, blood pressure, anthropometric measures, and blood samples were collected and analyzed. CAVI was measured using VaSera VS-1500 N devise. Subjects with a history of CVD or chronic renal disease were excluded.

**Results:**

One thousand nine hundred thirty-four participants, 44.7% of males, were included. The median age was 48 (Interquartile Range [IQR] 19) years, TG levels were 1.05 (0.793) mmol/L, and CAVI 7.24 (1.43) points. Prevalence of high CAVI was 10.0% (14.5% in males and 6.4% in females; *P* <  0.001) and prevalence of hypertriglyceridemia was 20.2% (29.2% in males and 13% in females, *P* <  0.001). The correlation between TG and CAVI was 0.136 (*P* <  0.001). High CAVI values were more prevalent among participants with metabolic syndrome (MetS), high blood pressure, dysglycemia, abdominal obesity, high LDL-cholesterol (LDL-c), and high total cholesterol. Using binary regression analysis, high TG were associated with high CAVI, even after adjustment for other MetS components, age, gender, smoking status, LDL-c, and statin treatment (β = 0.474, OR = 1.607, 95% CI = 1.063–2.429, *P* = 0.024).

**Conclusion:**

TG levels were correlated with ArSt, measured as CAVI. High TG was associated with high CAVI independent of multiple cardiometabolic risk factors. Awareness of the risks and targeted treatment of hypertriglyceridemia could further benefit in reducing the prevalence of CVD and events.

## Background

Cardiovascular disease (CVD) was the leading cause of mortality worldwide in 2017, with 17.9 million deaths, representing 31.8% of deaths worldwide and 49.1% in Central Europe [1], demanding an urgent call for preventive strategies, including the development of novel biomarkers related to arteriosclerosis for the early detection of possible atherosclerosis. Arterial stiffness (ArSt) is a predictor of cardiovascular events and mortality, independent of traditional risk factors [2, 3]. ArSt describes structural changes in arterial wall related to the loss of elasticity and is a strong predictor of CVD events and all-cause mortality [2, 3]. Measuring ArSt is one of the methods of quantitative estimation of arteriosclerosis extent [4], and can be measured both invasively and non-invasively. Pulse wave velocity (PWV) is considered the “gold standard” method to assess ArSt [4, 5].

A pulse wave moves along the arterial tree faster in the stiffer tube than in the elastic one. Nevertheless, at the moment of measurement, PWV is affected by acute blood pressure (BP) changes [2], e.g., stress-induced rise in BP can lead to the overestimation of PWV. The cardio-ankle vascular index (CAVI) may eliminate this limitation, by adjusting the formula for BP value [4]. Experimental studies have shown that administration of α1 and β1 adrenoreceptor blockers to reduce BP does not change CAVI values despite the significant and acute changes in BP [6]. The majority of the studies used PWV as a biomarker of ArSt [2–4, 6–9], but CAVI is easy to measure and is suitable for clinical use [4, 10]. Studies indicate that CAVI might be a more accurate ArSt parameter, in comparison with PWV [2, 7].

Metabolic syndrome (MetS) is a cluster of cardiometabolic risk factors including abdominal obesity, high BP, impaired blood glucose, high triglycerides (TG), and low high-density lipoprotein cholesterol (HDL-c) [11]. The presence of MetS has been related to high CAVI as a surrogate of ArSt [12–14], a higher number of MetS components was associated with higher CAVI values [12]. When MetS components were analyzed individually, high BP [12, 15–18], impaired blood glucose [12, 13, 15–17], abdominal obesity [14, 15, 19], and HDL-c [13, 14, 17, 20], were associated with ArSt, however, the association between high TG and ArSt shows contradictory results. Many studies report no association between high TG and ArSt [12–14, 17–20], and many others report a positive independent association between them [8, 9, 15, 21–25]. Most of these studies are not evaluating randomly selected population-based samples [8, 12, 13, 15, 19, 20, 23], mostly including high-risk individuals [9, 17, 18, 21], older age groups [9, 15, 19, 21, 24], and adjusting for components that are not specifically designed to evaluate the association between both parameters [13, 15, 19, 23, 24]. According to the best of our knowledge, none previous analysis was focused specifically on providing the answer to the question if high TG are associated with ArSt. This analysis aims to evaluate the association between high TG and ArSt, measured by CAVI, adjusting for MetS components and other related traditional risk factors, in a random population-based sample of Czech adults, living in Brno.

## Methods

### Design and population

The study design, sampling, and implementation were described in detail previously [26]. In brief, Kardiovize study is a prospective population-based study, with a sample of 25 to 64 years old Brno residents. The recruitment and core baseline examinations were completed in 2014. Follow-up will be executed regularly at 5-year intervals. It is anticipated that the study will run until 2030 [26].

Brno is the second-largest city in the Czech Republic. In January 2013, the population of Brno comprised of 373,327 residents [27]. The study enrolled 1% of the adult population of Brno in December of 2014. The study population was randomly selected and stratified by sex and age [26].

### Sampling and recruitment

**Fig. 1:**
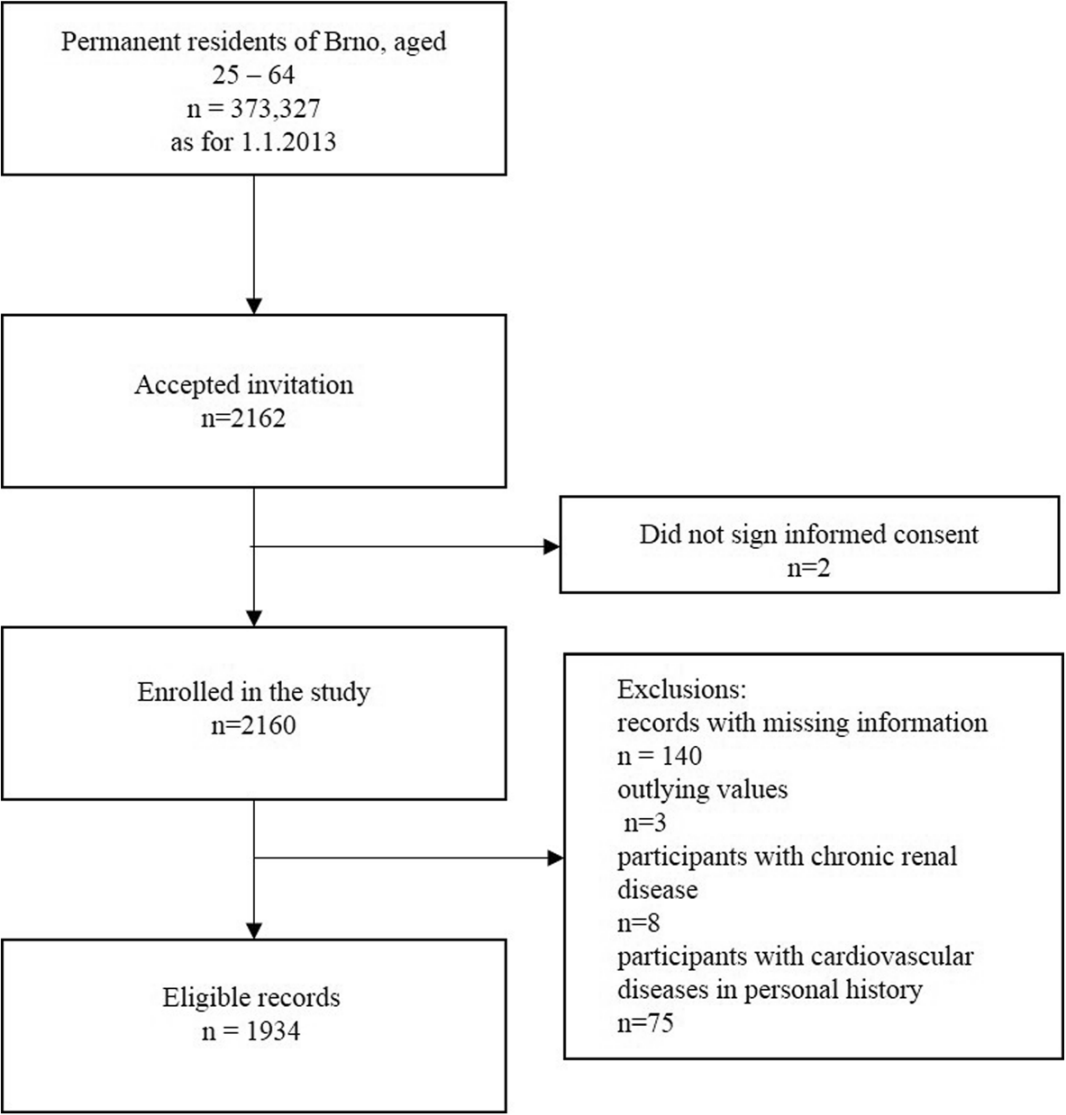
Flow chart of the recruitment, baseline data collection, and selection of participants for the analysis

Two survey samplings were carried out, using the registries of all health insurance companies, except one that declined to cooperate (representing 8.9% of the population). Via mail potential participants were informed about the study, it’s goals and the confidentiality of any provided information. Based on the two samplings with a total of 6377 randomly selected invitees, the overall response rate was 33.9%. Response rate differed among age groups, comprising 19.8, 30.6, 32.44, 40.4% among 25–34 years-old, 35–44 years-old, 45–54 years old and 55–64 years old respectively. Due to confidentiality restrictions, it was impossible to obtain any information on non-respondents [26]. A total of 2160 individuals were enrolled in the study and 226 records were excluded from the present analysis due to either missing information on ArSt or MetS components, or presence of the self-reported history of CVD, defined as stroke, chronic ischaemic disease, or chronic renal disease. Figure 1 summarizes the recruitment of the participants and further exclusion of non-eligible records.

### Data collection

Participants’ health was assessed by trained research nurses and physicians at the International Clinical Research Center of the St Anne’s University Hospital in Brno, using face-to-face interviews and comprehensive questionnaires. Collected data were stored in the web-based research electronic data capture (REDCap) database. The questionnaires included demographics, smoking, and socioeconomic status, diet, and alcohol consumption, physical activity, family and personal history, medications, and mental health. CAVI was measured using the VaSera VS-1500 N device (Fukuda Denshi Co., Ltd., Japan). Modular SWA P800 analyzer (Roche, Basel, Switzerland) was used to analyze the 12-h fasting blood samples. TG, total cholesterol, and glucose levels were measured by an enzymatic colorimetric technique (Roche Diagnostics GmbH, Germany). HDL-c was assessed by the homogeneous technique for direct measuring without precipitation (Sekisui Medical, Japan). If triglyceride levels were lower than 4.5 mmol/L, low-density lipoprotein cholesterol (LDL-c) was calculated according to the Friedewald equation only; if triglycerides were higher, LDL-c was measured by the homogeneous technique for direct measuring (Sekisui Medical, Japan). BP was measured, using an automated office measurement device (BpTRU, model BPM 200; Bp TRU Medical Devices Ltd., Canada). Self-reported smoking status was verified by measuring the amount of carbon monoxide in the expired breath, using Smokelyzer (Micro Smokerlyzer; BedFont Scientific Ltd., UK). The height and weight measurements were performed with a medical digital scale with a stadiometer (SECA 799; SECA, GmbH and Co. KG, Germany). The waist, hip, and neck circumferences were measure manually, using tape [26].

### Definition of variables

The presence of cardiometabolic risk factors was defined according to the MetS definition [11]. Hypertriglyceridemia was defined as TG level ≥ 1.7 mmol/l, or treatment with fibrates or nicotinic acid; low HDL-c level as HDL-c < 1 mmol/l in males and < 1.3 mmol/l in females, or treatment with fibrates or nicotinic acid; dysglycemia as previously diagnosed diabetes mellitus, present treatment of elevated glucose, or fasting plasma glucose ≥5.6 mmol/l; high BP as systolic ≥130 mmHg and/or diastolic ≥85 mmHg or treatment of elevated BP; the presence of abdominal obesity, identified as high waist circumference ≥ 94 cm in males and ≥ 80 in females. MetS was defined as a simultaneous presence of 3 or more of those risk factors [11].

Total cholesterol ≥5.2 mmol/l was considered high [28]. High LDL-c was categorized according to the global cardiovascular risk calculated by the SCORE and the LDL-c value: Low risk: LDL-c ≥ 3.0 mmol/L, moderate risk: LDL-c ≥ 2.6 mmol/L, high risk: LDL-c ≥ 1.8 mmol/L, very high risk: LDL-c ≥ 1.4 mmol/L [29].

CAVI values were derived from the β coefficient***,*** obtained from the Bramwell-Hill equation:

$$ \beta =2\rho\ \frac{1}{Ps- Pd}\ \mathit{\ln}\frac{Ps}{Pd}\  PWV2 $$

Where PWV is pulse wave velocity, ρ is blood density, Ps and Pd are systolic and diastolic BP values in mmHg [4]. High CAVI group was defined as those subjects with ≥9 points and normal as those with CAVI < 9, based on the presence of advanced arteriosclerosis [4–6, 30–34]. Levels of carbon monoxide in the expired breath < 10 ppm were compatible with the definition of a non-smoker. Smoking status was divided into 2 groups. Non-smokers were defined as those who have smoked less than 100 cigarettes in his/her life, or those, who have stopped smoking at least 1 year before the examination. Smokers were defined as those smoking either daily or less than daily in the past year, or those whose Smokelyzer values were ≥ 10 [26].

### Statistical analysis

Analyses were performed using the SPSS software (SPSS, version 23.0, Armonk, NY: IBM Corp.). Kolmogorov-Smirnov test was conducted to assess the normality of the continuous variables. Variables were non-normally distributed and presented as median (interquartile range), their differences were evaluated using the Mann-Whitney U test. Correlation Spearman analysis between TG and CAVI was assessed. Proportions were presented as percentages and differences were determined by the χ^2^ test. Univariate analysis was used to assess risk factors related with CAVI as a binary outcome (High ≥9 or normal < 9) and was presented as odds ratio (OR) and 95% confidence interval (CI). Assumptions of linear regression analysis were not met. Instead, binary regression analysis was done using CAVI as a binary outcome and TG as a dichotomic variable (high ≥1.7 mmol/l and normal < 1.7 mmol/l), and adjusted by multiple confounders (See Directed Acyclic Graph in Supplementary files) creating different models. The first model was adjusted by age and gender, and subsequent models by high waist circumference, elevated fasting glucose, systolic and diastolic BP, HDL-c, LDL-c, smoking status, total cholesterol, and statin treatment. In the multicollinearity test with CAVI as an outcome, the values of the variance inflation factors in these models were < 4.0. Statistical significance was considered as *P* <  0.05.

## Results

### Subject characteristics

In total 1934 participants were included (Fig. 1). Participants were 44.7% males, the median (IQR) age was 48 (19) years. Median (IQR) of TG and CAVI were 1.0 (0.7) and 7.2 (1.4), respectively. High BP, dysglycemia, hypertriglyceridemia, high CAVI, and high LDL-c were more prevalent in men, but high total cholesterol was more prevalent in women. The prevalence of smokers, abdominal obesity, and low HDL-c were similar between genders (Table 1). The prevalence of MetS was 23.2% (28.9% in males and 18.7% in females; *P* <  0.001). The prevalence of hypertriglyceridemia was 20.2% (29.2% in males and 13% in females; *P* <  0.001) (Table 1). Subjects with high triglycerides were older, had higher BP, fasting plasma glucose, LDL-c, total cholesterol, waist circumference, and CAVI than those with normal triglycerides *(*Table 2*)*. The prevalence of high CAVI was 10.0%, higher in men than women, 14.5, and 6.4% (*P* <  0.001), respectively (Table 2). Subjects with high CAVI were more likely to have MetS (OR = 3.6, 95% CI 2.70–4.96), high TG (OR = 2.5, 95% CI 1.86–3.51), abdominal obesity (OR = 2.2, 95% CI 1.58–3.06), dysglycemia (OR = 3.5, 95% CI 2.58–4.98), high BP (OR = 7.0, 95% CI 4.81–10.19), high LDL-c (OR = 3.5, 95% CI 2.37–5.24) and high total cholesterol (OR = 1.7, 95% CI 1.32–2.43). High CAVI was more prevalent in males (OR = 2.4, 95% CI 1.80–3.34) and increased with age. Among others, age older than 45 years and elevated BP were the risk factors associated with the higher chance of high CAVI. Smoking status and low HDL-c were not related to high CAVI (Table 3).

**Table 1:** Characteristics of subjects by gender

Variables	All	Male	Female	p
n (%)	1934 (100.0)	864 (44.7)	1070 (56.3)	
Age (years)	48.0 (19.0)	46.5 (20.0)	49.0 (20.0)	0.002
Systolic blood pressure (mmHg)	118.4 (19.6)	121.2 (17.6)	115.6 (19.4)	< 0.001
Diastolic blood pressure (mmHg)	79.4 (12.6)	82.2 (12.0)	77.0 (11.8)	< 0.001
Fasting plasma glucose (mmol/L)	4.9 (0.7)	5.0 (0.7)	4.8 (0.7)	< 0.001
Triglycerides (mmol/L)	1.0 (0.7)	1.2 (0.9)	0.9 (0.6)	< 0.001
HDL – Cholesterol (mmol/L)	1.4 (0.5)	1.3 (0.3)	1.6 (0.4)	< 0.001
LDL-Cholesterol (mmol/l)	3.0 (1.2)	3.0 (1.2)	2.9 (1.2)	0.051
Waist Circumference (cm)	88.0 (20.0)	95.0 (17.0)	82.0 (18.0)	< 0.001
CAVI	7.2 (1.4)	7.3 (1.5)	7.1 (1.3)	< 0.001
Total Cholesterol (mmol/L)	5.1 (1.3)	5.0 (1.3)	5.1 (1.3)	0.007
MetS (%)	449 (23.2)	249 (28.9)	200 (18.7)	< 0.001
High Blood pressure (%)	828 (42.8)	436 (50.5)	392 (36.6)	< 0.001
Dysglycemia (%)	290 (15)	171 (19.8)	119 (11.1)	< 0.001
Hypertriglyceridemia (%)	391 (20.2)	252 (29.2)	139 (13.0)	< 0.001
Low HDL-Cholesterol (%)	254 (13.1)	100 (11.6)	154 (14.4)	0.078
Abdominal obesity (%)	1093 (56.5)	484 (56.0)	609 (56.9)	0.712
High CAVI (%)	194 (10.0)	125 (14.5)	69 (6.4)	< 0.001
High Total Cholesterol (%)	914 (47.3)	379 (43.9)	535 (50.0)	0.008
High LDL-Cholesterol (%)	1204 (62.3)	593 (68.6)	611 (57.1)	< 0.001
Smokers (%)	467 (24.3)	220 (25.7)	247 (23.2)	0.200

Median (Interquartile range) and differences were assessed using the Mann-Whitney U test, n (%), and differences were assessed using the Chi-Square test

**Table 2:** Characteristics of subjects by the level of triglycerides

Variables	All	Normal triglycerides	High triglycerides	*P*
n (%)	1934 (100.0)	1543 (79.8)	391 (20.2)	
Male Gender	864	612 (70.8)	252 (29.2)	< 0.001
Female Gender	1070	931 (80.7)	139 (13.0)	
Age (years)	48.0 (19.0)	47.0 (19.0)	52.0 (18.0)	< 0.001
Systolic blood pressure (mmHg)	118.4 (19.6)	116.8 (18.6)	124.8 (20.6)	< 0.001
Diastolic blood pressure (mmHg)	79.4 (12.6)	78.2 (12.2)	83.4 (11.8)	< 0.001
Fasting plasma glucose (mmol/L)	4.9 (0.7)	4.8 (0.7)	5.0 (0.8)	< 0.001
Triglycerides (mmol/L)	1.0 (0.7)	0.9 (0.5)	2.1 (0.7)	< 0.001
HDL – Cholesterol (mmol/L)	1.4 (0.5)	1.5 (0.4)	1.2 (0.3)	< 0.001
Waist Circumference (cm)	88.0 (20.0)	86.0 (19.0)	99.0 (14.0)	< 0.001
CAVI	7.2 (1.4)	7.2 (1.3)	7.4 (1.8)	< 0.001
Total Cholesterol (mmol/L)	5.1 (1.3)	5.0 (1.2)	5.6 (1.3)	< 0.001
LDL - Cholesterol	3.0 (1.2)	2.9 (1.1)	3.3 (1.2)	< 0.001

Data are presented as median (Interquartile range) and differences were assessed using the Mann-Whitney U test

**Table 3:** Prevalence of high CAVI and risk factors related using a Univariate Analysis

	High CAVI (%)	OR	95% CI	*P*
All subjects	10.0			
Age 25–44/Age 45–64	1.1 /17.1	19.2	9.78–37.79	< 0.001
Male/Female	14.5 /6.4	2.4	1.80–3.34	< 0.001
MetS	20.9	3.6	2.70–4.96	< 0.001
Without MetS	6.7	1.0		
High TG	18.2	2.5	1.86–3.51	< 0.001
Normal TG	8.0	1.0		
Abdominal Obesity	12.9	2.2	1.58–3.06	< 0.001
Without Abdominal obesity	6.3	1.0		
Dysglycemia	23.1	3.5	2.58–4.98	< 0.001
Without Dysglycemia	7.7	1.0		
Low HDL-c	10.2	1.0	0.66–1.58	0.911
Normal HDL-c	10.0	1.0		
High BP	19.1	7.0	4.81–10.19	< 0.001
Normal BP	3.3	1.0		
Smoker	12.0	1.3	0.94–1.80	0.133
Non-Smoker	9.5	1.0		
High TC	12.8	1.7	1.32–2.43	< 0.001
Normal TC	7.5	1.0		
High LDL-c	13.5	3.5	2.37–5.24	< 0.001
Normal LDL-c	4.2	1.0		

Abbreviations: *OR* odds ratio, *CI* confidence interval, *MetS* metabolic syndrome, *TG* triglycerides, *HDL-c* HDL cholesterol, *BP* blood pressure, *TC* total cholesterol. Proportions were presented as percentages and differences were determined by the χ^2^ test. Univariate analysis was used to assess risk factors related with CAVI as a binary outcome (High ≥9 or normal < 9) and was presented as OR and 95% CI. Risk factors are presented as dichotomic variables

### Associations between CAVI and triglycerides

**Fig. 2:**
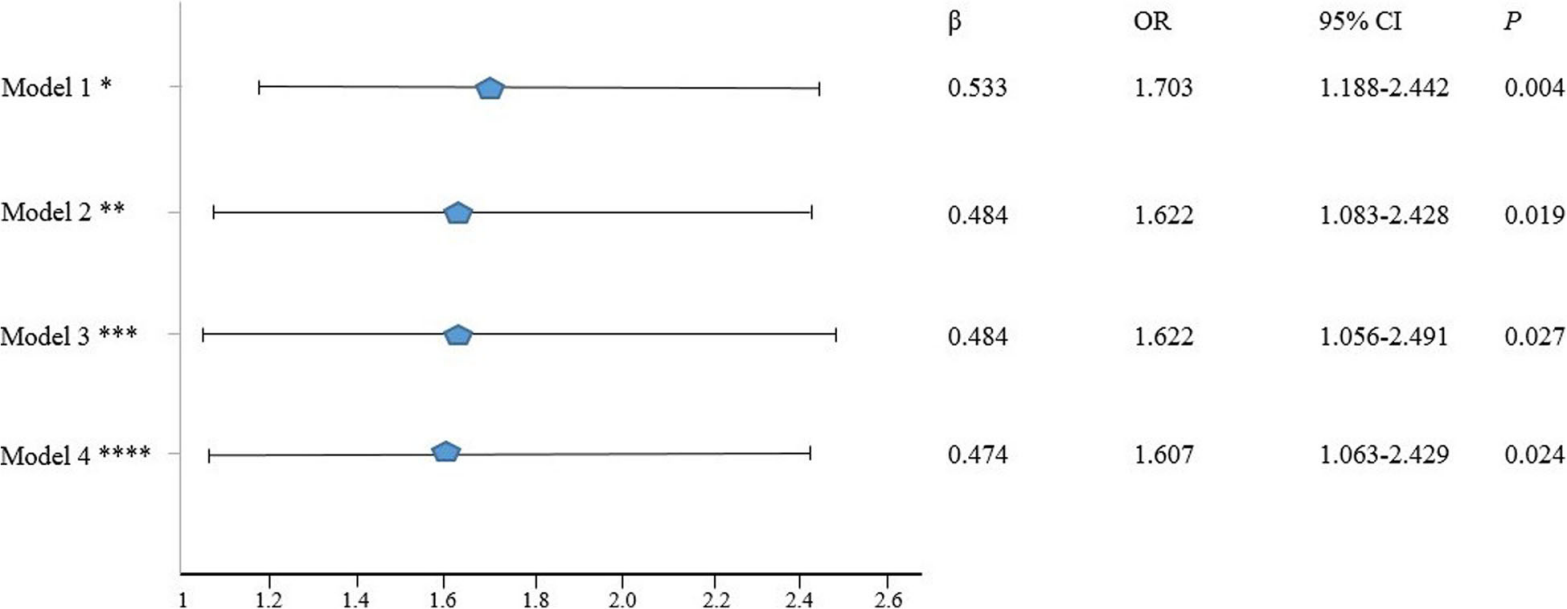
Binary regression models associating high TG ≥ 1.7 mmol/l and high CAVI ≥9. * adjusted by age and gender. ** adjusted by age, gender, high waist circumference ≥ 80 cm in females and ≥ 94 cm in males, elevated fasting glucose ≥5,6 mmol/l or treatment, systolic and diastolic BP levels, HDL-c values. *** as model 2, further adjusted by smoking status and TC levels. **** as model 2, further adjusted by smoking status, LDL-c levels and statin treatment. Abbreviations: BP – blood pressure, CAVI – cardio-ankle vascular index, CI – confidence interval, HDL-c – HDL cholesterol, LDL-c – LDL-cholesterol, OR – Odds ratio, TC – total cholesterol, TG – triglycerides

TG and CAVI were significantly and positively correlated (r = 0.136; *P* <  0.001). Further, four models were created to assess the independent association between high TG and high CAVI (Fig. 2). In model 1, adjusting by age and gender, subjects with high TG had 70% higher odds of having high CAVI than those with normal TG (β = 0.533, OR = 1.703, 95% CI = 1.188–2.442). In model 2, adjusted by age, gender, and individual components of MetS the odds of having high CAVI were 62% (β = 0.484, OR = 1.622, 95% CI = 1.083–2.428). In model 3, adding smoking status and total cholesterol to model 2, the odds of having high CAVI remained 62% (β = 0.484, OR = 1.622, 95% CI = 1.056–2.491). Finally, in model 4, adding smoking status, LDL-c, and statin treatment to model 2, the odds of having high CAVI were 60% (β = 0.474, OR = 1.607, 95% CI = 1.063–2.429). In this model, LDL-c was not associated with high CAVI (β = − 0.174, OR = 0.840, 95% CI = 0.682–1.035, *P* = 0.102).

## Discussion

To the best of our knowledge, this may be the first analysis specifically designed to address the question if high TG are associated with ArSt, where previous reports show contradictory results. Using a randomly selected large population-based sample, high TG (≥ 1.7 mmol/l) increased the odds of having high CAVI (≥9) by 60%, independent of multiple confounding variables as age, gender, MetS components, LDL-c, statin treatment, and smoking habits. Prevalence of high CAVI was 10.0% and was associated with male gender, higher age, high BP, LDL-c and total cholesterol, presence of dysglycemia, and abdominal obesity, but not related to smoking status and low HDL-c.

Consistent with this result, in Japan [23], in 23,257 urban residents, aged 47.1 ± 12.5 years, odds of having a high CAVI (≥90th percentile) per 1-standard deviation increment of Log_e_TG were almost double (OR = 1.9, 95% CI = 1.81–1.99). In this population, a cut-off value of TG 1.05 mmol/l was more sensitive and specific predicting high CAVI (OR = 2.43, 95 CI 2.14–2.75) than the threshold, currently used in clinical practice (1.7 mmol/l) [11, 23]. In China [8], in 16,733 adults from the southern part of the country, aged 18 or older, subjects with high TG (≥1.7 mmol/l) and LDL-c below 1.8 mmol/l were 144% times more likely to have high PWV in comparison to subjects with normal TG and low LDL-c (OR = 2.44, 95% CI = 1.61–3.71) [8]. In 14,071 hypertensive patients from Jiangsu and Anhui Provinces of China [9], with the mean age of 64.4 ± 7.4 years, the association between TG and PWV stayed significant even after adjusting the results for gender, age, BP, BMI, fasting blood glucose, medical treatment, smoking, alcohol consumption, etc. (β = 0.54, 95% CI = 0.44–0.65, *P* <  0.001) [9]. In a prospective observational study assessing 1447 community-based residents from Beijing [24], followed by 4.8 years, baseline TG was strongly correlated with ArSt during the follow-up evaluation (carotid-femoral PWV; β = 0.747, 95% CI = 0.394–1.100, *P* <  0.001 and carotid-radial PWV; β = 0.367, 95% CI = 0.140–0.593, *P* = 0.002). Moreover, changes in TGs were directly associated with changes in PWV, every standard deviation increase in TG levels between baseline and follow-up was linked to 29% higher risk for increased change in the PWV between baseline and follow-up (OR = 1.296, 95% CI = 1.064–1.580, *P* = 0.010) [24]. In Spain [15], in 2351 subjects with the mean age of 61.4 ± 7.7, all MetS components, except HDL-c, were associated with CAVI. TG values were significantly and positively related to CAVI using multiple linear regressions models (β = 0.001, 95% CI = 0.001–0.001; *P* = 0.002) [15].

On the contrary, in 18 countries from Europe [19], assessing 2224 subjects, aged 40 and older, PWV was higher in subjects with MetS compared with those without (9.57 ± 0.06 vs 8.65 ± 0.10; *P* <  0.001), but CAVI was similar in those two groups (8.34 ± 0.03 vs 8.29 ± 0.04; *P* = 0.40). In the multiple regression analysis, PWV was positively associated with age, BP, glucose, and HDL-c, but not with waist circumference and TG; CAVI was positively associated with age, gender, BP, glucose, but not with TG and HDL-c, and negatively associated with waist circumference. Authors don’t provide a clear explanation with contradictory results relating to waist circumference and CAVI [19]. In a prospective evaluation of 2106 middle-aged subjects with MetS from Lithuania [17], high CAVI values at the baseline were related to a higher risk for CVD events after around 4 years. At the baseline, high CAVI values were related to worse cardiometabolic profile, but not to TG value (*P* = 0.891) [17]. In Korea, in 1144 adults, older than 18 years from Gyeonggi [12], assessing the association between MetS and CAVI, CAVI was independently related with age, sex, diastolic BP, and uric acid, but not with waist circumference, plasma glucose, HDL-c, and TG [12], In two Chinese population-based studies [13, 20], TG were significantly correlated with CAVI, but this association disappeared after multiple adjustments.

LDL-c has not shown the independent statistically significant association with CAVI in the present analysis after adjustment for potential confounders (*P* = 0.102). These results are consistent with the meta-analysis of Željko et al. [35], which included 8 studies, involving 317 patients with familial hypercholesterolemia and 244 healthy controls, suggesting no difference between PWV between the groups (Weighted mean difference (WMD): 0.17 m/s, 95% CI: − 0.31, 0.65, *P* = 0.489; I^2^ = 80.15%).

Discrepancies between results of the studies might be partially explained by the differences in the population sample sizes, inclusion criteria for the subject’s recruitment, way of ArSt quantification, or the variety of adjustment variables. Studies, performed on large population samples tended to observe a positive association between ArSt and TG, however, some of them used PWV as an ArSt marker [8, 9, 24]. Also, studies that failed to find an association between TG and ArSt were often conducted on the population samples with MetS [17, 19] or diabetes [18], meanwhile, studies that are indicating positive relationship included mostly healthy subjects [9, 22, 25]. More prospective studies are needed, to clarify the risk of elevated TG and its’ effect on the arterial wall state.

Most commonly, therapy of dyslipidemia is focused on lowering LDL-c levels. Non-HDL-C (representing the sum of cholesterol in LDL, intermediate-density lipoprotein [IDL], and very-low-density lipoprotein [VLDL] particles) is a preferred secondary treatment target. However, therapy focused only on LDL-c will not address other abnormalities of lipid spectrum (as high levels of TG and low HDL-c levels), which increase residual cardiovascular risk, even after reaching recommended levels of LDL-c [36, 37].

The whole spectrum of possible underlying pathophysiological mechanisms of the influence of lipid profile on ArSt has not been well established yet. However, abnormal lipid profile simultaneously influences several pathways – the development of atherosclerotic plaques, oxidative stress, inflammation enhancement, endothelial dysfunction, and low availability of nitric oxide [38]. From the point of view of atherosclerosis and CVD, there are four main mechanisms which can indirectly increase CVD risk. First, hydrolysis of postprandial chylomicrons or endogenously formed VLDL leads to further formation of cholesterol-rich remnants, which can enter the subendothelial space through the scavenger receptors and promote the formation of the foam-cells [23]. Second, higher Apolipoprotein (Apo) CIII might also have an impact on the metabolism of TGs, through inhibition of TG hydrolysis and increased formation of dense, oxidation-prone low-density lipoprotein particles [9, 39]. Liver fat mass was also directly associated with the amount of secreted very low-density lipoprotein [39]. Third, high TG might disrupt the mechanism of reverse cholesterol transport [39]. Fourth, in vitro analysis indicates that high TG might also promote endothelial dysfunction, by stimulating the expression of endothelial mediators, such as endothelin-1 [23].

One of the potential confounders in the relationship between TG and ArSt is treatment with statins, which are one of the most commonly prescribed drugs. Statins have shown diverse effects on the human body, including improving endothelial function in the presence of atherosclerotic risk factors. A recent meta-analysis [40] included 18 controlled trials with 1701 subjects, aimed to assess the effects of statin therapy on ArSt showed a significant reduction of augmentation index independent on LDL-c changes (WMD: -2.40, 95% CI: − 4.59, − 0.21, *P* = 0.032; I^2^: 51.20%) [40].

### Strength and limitations of the study

The main strength of the study is the representative population-based sample and a wide spectrum of tests performed. The main limitation of the present report is that the cross-sectional design doesn’t allow to establish causality between TG and ArSt. Independent association between TG and CAVI as continuous variables was not reported because the assumptions of linear regression analysis were not met. The future prospective results of this study will allow us to examine the predictive value of lipid profiles on ArSt.

## Conclusion

TG levels were correlated with ArSt, measured as CAVI. High TG was associated with high CAVI independently of age, gender, presence of MetS components, smoking status, LDL-c, and statin treatment. This result highlights the negative influence of high TG in the process of atherosclerosis. Raising awareness of the risks and targeted treatment of hypertriglyceridemia could further benefit in reducing the prevalence of CVD and events.

## Supplementary information

**Additional file 1.** Directed Acyclic Graphs explaining the relationship of confounding variables with high triglycerides and arterial stiffness. Visual representation of the binary regression models (independent variable, dependent variable, and adjusting variables). Model 1: adjusted by age and gender. Model 2: adjusted by age, gender, high waist circumference ≥ 80 cm in females and ≥ 94 cm in males, elevated fasting glucose ≥5,6 mmol/l or treatment, systolic and diastolic BP levels, HDL values. Model 3: as model 2, further adjusted by smoking status and TC levels. Model 4: as model 2, further adjusted by smoking status, LDL-c levels, and treatment with Statins. Abbreviations: BP – blood pressure, HDL-c – HDL-cholesterol, LDL-c – LDL-cholesterol, TC – total cholesterol, TG – triglycerides.

## Data Availability

The datasets generated and analyzed during the current study are available from the corresponding author on reasonable request.
