# Supplementary material for: Associations between high triglycerides and arterial stiffness in a population-based sample: Kardiovize Brno 2030 study
Source: Lipids Health Dis. 2020 Jul 15;19:170. doi: 10.1186/s12944-020-01345-0 (PMC7362636; doi:10.1186/s12944-020-01345-0)
Supplement: Supplementary file 1 — Additional file 1. Directed Acyclic Graphs explaining the relationship of confounding variables with high triglycerides and arterial stiffness. Visual representation of the binary regression models (independent variable, dependent variable, and adjusting variables). Model 1: adjusted by age and gender. Model 2: adjusted by age, gender, high waist circumference ≥ 80 cm in females and ≥ 94 cm in males, elevated fasting glucose ≥5,6 mmol/l or treatment, systolic and diastolic BP levels, HDL values. Model 3: as model 2, further adjusted by smoking status and TC levels. Model 4: as model 2, further adjusted by smoking status, LDL-c levels, and treatment with Statins. Abbreviations: BP – blood pressure, HDL-c – HDL-cholesterol, LDL-c – LDL-cholesterol, TC – total cholesterol, TG – triglycerides. [file 12944_2020_1345_MOESM1_ESM.docx]

Age

Arterial Stiffness

Gender

High TG

Model 1:

Age

Abdominal obesity

High BP

Arterial Stiffness

Gender

High TG

Dysglycemia

Low HDL-c

Model 2:

Total Cholesterol

Smoking

Age

High BP

Arterial Stiffness

Gender

High TG

Dysglycemia

Low HDL-c

Abdominal Obesity

Model 3:

Model 4:

Arterial Stiffness

Age

Gender

High TG

High BP

Dysglycemia

Low HDL-c

Abdominal Obesity

Total Cholesterol

Smoking

High LDL-c

Treatment with Statins

Figure: Directed Acyclic Graphs explaining the relationship of confounding variables with high triglycerides and arterial stiffness.
Model 1: adjusted by age and gender.
Model 2: adjusted by age, gender, high waist circumference ≥80 cm in females and ≥94 cm in males, elevated fasting glucose ≥ 5,6 mmol/l or treatment, systolic and diastolic BP levels, HDL values.
Model 3: as model 2, further adjusted by smoking status and TC levels.
Model 4: as model 2, further adjusted by smoking status, LDL-c levels and treatment with Statins.

Abbreviations: BP – blood pressure, HDL-c – HDL-cholesterol, LDL-c – LDL-cholesterol, TG – triglycerides.
